# Treatment Options for Metallo-Beta-Lactamase-Producing *Enterobacterales* in the Era of Increasing Resistance

**DOI:** 10.3390/ijms27125320

**Published:** 2026-06-12

**Authors:** Anastasia Golovina, Fedor Antipin, Igor Khalymbadzha, Olga Terina, Daniil Yakovlev, Elena Fedina, Roman Ivanov

**Affiliations:** Translational Medicine Research Center, Sirius University of Science and Technology, Olympic Ave. 1, Federal Territory “Sirius”, 354340 Sochi, Russia; antipin.fv@talantiuspeh.ru (F.A.); i.a.khalymbadzha@urfu.ru (I.K.); terinaolga13@gmail.com (O.T.); sirius.yakovlev@gmail.com (D.Y.); fedina.es@talantiuspeh.ru (E.F.); ivanov.ra@talantiuspeh.ru (R.I.)

**Keywords:** aztreonam–avibactam, cefiderocol, colistin, tigecycline, fosfomycin, ceftazidime–avibactam + aztreonam, metallo-beta-lactamase, *Enterobacterales*

## Abstract

The escalating global burden of antimicrobial resistance constitutes a public health crisis. The World Health Organization reports that epidemiological models project 10 million deaths attributable to this issue annually by 2050. Among resistant pathogens, metallo-β-lactamase (MBL)-producing organisms represent a clinical challenge, given their consistent association with high rates of morbidity and mortality. This review summarizes effective treatment options against MBL-producing *Enterobacterales*. In clinical practice, a pragmatic therapeutic decision rule can be applied: when aztreonam–avibactam (ATM–AVI) is accessible and in vitro susceptibility is confirmed, it should be regarded as the preferred targeted regimen for infections caused by MBL-producing *Enterobacterales*. In settings where ATM–AVI is unavailable, the combination of ceftazidime–avibactam with aztreonam (CAZ–AVI + ATM) remains the treatment of choice, an approach endorsed by current recommendations from the Infectious Diseases Society of America (IDSA) and the European Society of Clinical Microbiology and Infectious Diseases (ESCMID). Critical evaluation of the published evidence is essential to inform the selection of optimal therapeutic regimens for affected patients. Novel antimicrobial agents are currently under clinical development and may broaden the therapeutic toolkit in the near future.

## 1. Introduction

The World Health Organization 2024 Bacterial Priority Pathogens List reaffirms the critical importance of key Gram-negative pathogens, particularly members of the Enterobacteriaceae family [1,2,3,4,5]. Significantly, *Klebsiella pneumoniae* and *Enterobacter* spp. remain classified as critical priorities owing to their high mortality rates and extensive drug resistance profiles [6,7,8]. As part of the ESKAPE group, these bacteria possess the ability to ‘escape’ standard antibiotic therapies. Their classification as critical priority pathogens highlights both their substantial contribution to the antimicrobial resistance crisis and the urgent need for the development of novel therapeutic agents targeting these species. Resistance among these organisms encompasses multiple major antibiotic classes, including carbapenems, cephalosporins, β-lactams, aminoglycosides, and fluoroquinolones [9,10]. Isolates resistant to at least three of these classes are defined as multidrug-resistant (MDR), significantly limiting available treatment options.

In response to the urgent need for effective therapeutic options, two principal strategies have been proposed. The first involves the development of multitargeting antibiotics—single agents capable of simultaneously inhibiting multiple bacterial targets—while the second focuses antimicrobials directed against evolutionarily conserved and functionally constrained bacterial components [11,12]. In this context, the present study evaluates potential antibiotic therapies for infections caused by MBL-producing GNB, a clinical setting characterized by limited treatment options, based on an analysis of relevant clinical data.

**Figure 1:**
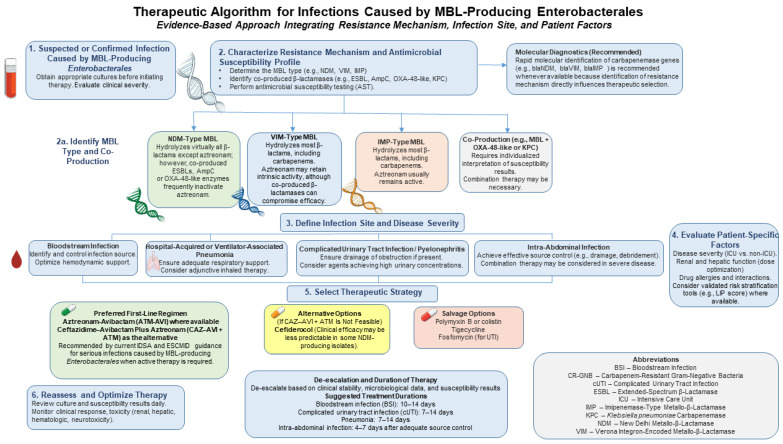
Therapeutic Algorithm for Infections Caused by MBL-Producing *Enterobacterales*.

We reviewed published cost studies of regimens active against MBL-producing *Enterobacterales* [13,14,15,16]. Because the studies differed in country, currency year, payer perspective, and cost components, the data were not pooled into a single standardized model. Renal dose adjustment is clinically relevant for several of these agents but was not consistently reported, further limiting cross-study comparisons. In general, colistin-based therapy was the least expensive, whereas novel β-lactams were the most expensive; however, these cost patterns should be interpreted cautiously and should not replace local formulary pricing. When total treatment costs, including hospitalization and adverse-event management, were considered, the differences between groups were smaller. A treatment algorithm incorporating these considerations is provided in Figure 1. To facilitate clinical application of the available evidence, Figure 1 presents a therapeutic algorithm integrating resistance mechanism (e.g., NDM-, VIM-, and IMP-type MBLs), infection site, disease severity, and patient-specific factors. The algorithm is intended to support bedside decision-making and antimicrobial stewardship when selecting treatment for MBL-producing Gram-negative infections.

## 2. Cefiderocol

Cefiderocol (CFDC) is a first-in-class, injectable siderophore cephalosporin that incorporates a catechol moiety, which facilitates iron-mediated active transport across the bacterial outer membrane [17]. Cefiderocol binds extracellular iron to form a chelate complex that is transported into the bacterial cell. After translocation into the periplasmic compartment, it exerts bactericidal activity through high-affinity binding to penicillin-binding proteins, thereby disrupting the final stages of peptidoglycan synthesis [18]. The C-3 side chain of cefiderocol is similar to that of cefepime, which enhances solubility and resistance to β-lactamase-mediated degradation. In November 2019, the FDA approved CFDC for treating complicated urinary tract infections (cUTIs) in adults with limited treatment options [19]. In April 2020, the European Medicines Agency (EMA) approved CFDC for the treatment of aerobic GNB infections in adults with limited therapeutic options. Subsequently, FDA approved the drug for hospital-acquired and ventilator-associated bacterial pneumonia (HABP/VABP) in September 2020, followed by EMA approval for these same indications [20].

Cefiderocol has demonstrated potent in vitro activity against multidrug-resistant pathogens, including *Enterobacterales*, non-fermenting GNB, and carbapenemase-producing strains [21,22,23]. In the CREDIBLE-CR trial, *K. pneumoniae* was identified in 39 patients (33%). The cefiderocol MIC_90_ value for carbapenem-resistant (CR) *K. pneumoniae* was 4 µg/mL. Among patients with carbapenem-resistant *Enterobacterales* (CRE) infections, which included *K. pneumoniae*, a numerically higher proportion achieved clinical cure in the cefiderocol group (66%) than in the best available therapy group (45%). However, reports suggest that MBL-CPE demonstrates resistance to CFDC, with the mechanisms contributing to this resistance including a heightened copy number of blaNDM genes [24] and alterations affecting the absorption of siderophores, including the CirA receptor [25].

Non-inferiority of cefiderocol versus standard of care was demonstrated in patients with CR bloodstream infections (BSIs). Mortality outcomes were comparable, with a 14-day all-cause mortality rate of 14% (9/64) in the cefiderocol group and 10% (6/63) in the control group [26].

A post hoc analysis of the phase 3 APEKS-NP and CREDIBLE-CR trials demonstrated that cefiderocol achieved a higher clinical cure rate (73.3%) compared to comparator antibiotics (20%) for infections caused by MBL-producing CRE [27]. However, efficacy concerns remain regarding CFDC use against MBL-producing strains, particularly those producing NDM. NDM-producing CRE often exhibit CFDC minimum inhibitory concentration (MIC) values near the EUCAST susceptibility breakpoint, with only 41% of isolates inhibited at 2 µg/mL [28]. European surveillance data (SIDERO-CR 2014–2016) reported cefiderocol susceptibility in 79.0% of VIM-producing and 51.4% of NDM-producing isolates [29]. Furthermore, a large cohort study found that only 33.2% of MBL-producing CRE were fully susceptible to cefiderocol, while most isolates fell within the area of technical uncertainty (ATU) according to the 2023 EUCAST criteria [30].

The predominant resistance mechanism involved impaired influx mediated by iron transporters. Although CFDC exhibits potent in vitro activity against GN pathogens and currently low rates of clinical resistance, the potential for resistance emergence remains a concern, particularly in the context of increasing and insufficiently monitored use. Therefore, careful antibiotic use, effective surveillance and stewardship programs, and resistance testing to assess CFDC efficacy are essential [31].

## 3. Tigecycline

Tigecycline is a last-resort intravenous glycylcycline antibiotic indicated for severe infections, including complicated skin and soft tissue infections and complicated intra-abdominal infections (cIAIs). Its clinical value derives from its efficacy against multidrug-resistant (MDR) pathogens when other therapeutic options are exhausted, as it overcomes key resistance mechanisms such as efflux pumps and ribosomal protection [32].

However, the clinical utility of tigecycline is tempered by significant safety concerns. A meta-analysis of 15 randomized controlled trials involving 7689 adults found a higher incidence of adverse events and all-cause mortality in patients receiving tigecycline compared to comparator antimicrobials. An analysis of trials across all approved indications revealed an adjusted mortality rate of 2.5% (66/2640) for tigecycline versus 1.8% (48/2628) for controls, with a risk difference of 0.6% (95% CI 0.0, 1.2) [33]. The underlying cause of this mortality discrepancy remains undetermined, although fatalities are generally attributed to infection progression or pre-existing comorbidities. Beyond the class-specific adverse events typical of tetracyclines, tigecycline has been associated with impaired liver function, pancreatitis, hypoglycemia, and an increased risk of mortality in hospital-acquired pneumonia (HAP) [34,35].

The efficacy of tigecycline varies substantially by infection site. For BSI caused by CRE, the standard dosage achieves low serum concentrations, raising concerns about its effectiveness. High-dose regimens (200 mg loading dose followed by 100 mg every 12 h) have been investigated to address this pharmacokinetic limitation. In a retrospective cohort study of 40 patients with carbapenem-resistant *Klebsiella pneumoniae* BSI in the intensive care unit, high-dose tigecycline was associated with significantly longer survival times (mean 83 days vs. 28 days; *p* = 0.027) and numerically lower in-hospital mortality (52.2% vs. 76.5%; *p* = 0.117) compared to standard-dose therapy, without an increase in adverse effects. Nevertheless, for complicated urinary tract infections (cUTIs), tigecycline is not approved due to limited urinary excretion and a lack of robust trial data, though it remains a last-resort option for carbapenem-resistant Gram-negative infections [36,37].

Data on combination therapy remain conflicting. A meta-analysis of mortality outcomes comparing colistin plus tigecycline versus either agent alone found no significant advantage for the combination: the odds ratio for 30-day mortality was 1.31 (95% CI 0.78–2.21) versus colistin monotherapy and 0.82 (95% CI 0.39–1.71) versus tigecycline monotherapy. However, a separate systematic review suggested that tigecycline monotherapy was associated with significantly higher mortality compared to tigecycline-containing combination regimens in patients with *Klebsiella pneumoniae* BSI (OR 2.86, 95% CI 1.46–5.59) [38].

A 2024 systematic review of *E. coli* BSI in patients with hematological malignancies, including hematopoietic stem cell transplantation (HSCT) recipients, reported very low tigecycline resistance, ranging from 0% to 8%, despite carbapenem resistance rates reaching up to 80% and ESBL production ranging from 13% to 80% [39]. Although MBL-specific susceptibility data were not reported separately, the high frequency of carbapenem resistance in this population suggests that tigecycline may still have a role as part of empirical combination therapy when MBL-producing *E. coli* is suspected, provided that its known safety limitations are carefully considered.

Notably, robust clinical evidence specifically evaluating tigecycline against infections caused by MBL-producing Enterobacteriaceae is lacking. Although early in vitro studies suggested near-universal susceptibility, recent isolates—particularly those co-harbouring multiple carbapenemase genes—exhibit variable susceptibility profiles. Consequently, any inference regarding the clinical utility of tigecycline in this context remains largely extrapolated from microbiological data and studies encompassing broader CR populations, in the absence of dedicated, MBL-stratified clinical outcomes.

Among newer tetracycline derivatives, omadacycline has demonstrated in vitro activity against some CRE, and a 2025 case report described its successful use in a patient with severe pneumonia caused by KPC-producing *Klebsiella pneumoniae* and *Acinetobacter baumannii* who had failed moxifloxacin and doxycycline [40]. It must be emphasized that this case involved serine-carbapenemase (KPC) production, not MBL production, and therefore these findings cannot be extrapolated to MBL-producing pathogens. Nevertheless, omadacycline could be considered as an alternative when tigecycline is unsuitable (e.g., due to intolerance, resistance, or drug interactions) and when MBL co-production has been excluded.

## 4. Ceftazidime–Avibactam + Aztreonam

Ceftazidime–avibactam (CAZ-AVI) is an intravenous combination of a third-generation antipseudomonal cephalosporin and avibactam, a non-β-lactam β-lactamase inhibitor. Avibactam inhibits Ambler class A (e.g., KPC), class C (AmpC), and certain class D (e.g., OXA-48) serine β-lactamases via a reversible covalent mechanism; however, it lacks activity against class MBLs [41,42]. Conversely, aztreonam (ATM) is stable against MBL hydrolysis but remains vulnerable to serine β-lactamases. Therefore, the combination of CAZ-AVI plus ATM is increasingly utilized for MBL-producing organisms: avibactam protects both ceftazidime and aztreonam from serine β-lactamase-mediated degradation, while aztreonam maintains activity against the MBL target [43].

Preclinical studies support this synergistic approach. In a cohort of 139 carbapenem-resistant *Enterobacterales* (CRE) isolates, CAZ-AVI plus ATM demonstrated in vitro susceptibility in 99.3% of cases [44]. However, a recent audit of 27 clinical NDM-producing isolates identified that while synergy was universal, the minimum inhibitory concentration (MIC) exceeded 1 mg/L in four isolates (19%), underscoring the need for susceptibility testing [45].

### Clinical Evidence and Interpretation

Clinical evidence supporting CAZ-AVI plus ATM is derived primarily from observational studies, which warrant cautious interpretation. A multicenter study by Falcone et al. involving patients with infections caused by *K. pneumoniae* resistant to all standard β-lactam/β-lactamase inhibitor combinations reported significantly lower 30-day mortality with CAZ-AVI plus ATM (19.2%) compared to best available therapy (BAT) (44%; *p* = 0.007) [46].

To mitigate biases inherent in observational research, a target trial emulation of 243 patients with MBL-producing *Enterobacterales* bacteremia reported lower 30-day mortality in the CAZ-AVI plus ATM group compared to other active antibiotics (35% vs. 47%; adjusted OR 0.63; 95% CI: 0.43–0.91; *p* < 0.01). While an E-value of 2.55 suggests the findings are robust against unmeasured confounding, such indicators do not eliminate the risk of residual bias. A smaller study of ICU patients with MBL-producing *K. pneumoniae* bacteremia reported a directionally similar but statistically non-significant mortality reduction (33% vs. 65%; OR 0.27) [47]. Overall, existing data suggest a consistent clinical benefit, though the strength of evidence is constrained by the lack of randomized controlled trials (RCTs).

In the absence of RCT data, a definitive first-line designation for CAZ-AVI plus ATM remains challenging. The 2024 IDSA guidance recommends CAZ-AVI plus ATM as a preferred option for MBL-producing *Enterobacterales* when ATM-AVI is unavailable, and the 2022 ESCMID guidelines similarly support this combination [3,4]. Future research should prioritize patient-level meta-analyses, standardized synergy testing, and molecular characterization of resistance mechanisms to better define the therapeutic benefit and identify patient subgroups most likely to derive a survival advantage [48,49].

## 5. Aztreonam–Avibactam

Aztreonam–avibactam (ATM-AVI) combines the monobactam aztreonam with the β-lactamase inhibitor avibactam. Aztreonam is inherently stable against metallo-β-lactamases (MBLs) but is susceptible to hydrolysis by co-produced serine β-lactamases, such as ESBLs, AmpC, and KPC. Avibactam inhibits these serine enzymes, protecting aztreonam and restoring its bactericidal activity against MBL-producing *Enterobacterales* [50].

ATM-AVI exhibits potent in vitro activity. In a large surveillance study (2019–2021) of 7446 *Enterobacterales* isolates, 1610 (21.6%) harbored MBL-encoding genes—predominantly NDM (*n* = 1544), followed by IMP (*n* = 28) and VIM (*n* = 27). ATM-AVI inhibited 98.8% of these MBL-positive isolates at ≤8 mg/L (MIC_90_ = 2 mg/L), with 100% susceptibility among VIM and IMP producers and 98.7% among NDM producers. Comparatively, other agents—with the exception of tigecycline (93.8% inhibited)—demonstrated limited activity against this population. Notably, only 0.2% of all screened isolates (61/24,937) exhibited an ATM-AVI MIC > 8 mg/L; among *E. coli* isolates with elevated MICs, 71.1% harbored a CMY-type AmpC enzyme [51].

### Clinical Evidence and Interpretation

Clinical data for ATM-AVI remain limited, derived from two randomized trials with small subsets of patients with confirmed MBL-producing infections. The Phase 3 ASSEMBLE trial compared ATM-AVI to best available therapy (BAT) for serious infections due to MBL-producing, multidrug-resistant Gram-negative bacteria. Within the microbiological intent-to-treat (micro-ITT) population, only 15 patients were evaluable (ATM-AVI: *n* = 12; BAT: *n* = 3). At the end of treatment, favorable microbiological response was 75.0% (9/12) with ATM-AVI versus 0% (0/3) with BAT; by the test-of-cure (Day 28), these rates were 50% (6/12) versus 0% (0/3), respectively. All-cause 28-day mortality was 8.3% (1/12) in the ATM-AVI group and 33.3% (1/3) in the BAT group [52]. While these findings suggest potential efficacy, the sample size is insufficient to draw definitive conclusions.

The REVISIT trial randomized 422 hospitalized adults with complicated intra-abdominal infection (cIAI) or hospital-acquired/ventilator-associated pneumonia (HAP/VAP) to ATM-AVI (plus metronidazole for cIAI) or meropenem (with or without colistin). Although *Enterobacterales* predominated (93%), only 10 patients had confirmed MBL-positive pathogens. In this subgroup, clinical cure rates at test-of-cure were numerically lower for ATM-AVI (28.6%; 2/7) than for meropenem (66.7%; 2/3). Overall 28-day mortality was low: 4% (12/282) for ATM-AVI and 7% (10/140) for meropenem. REVISIT trial does not provide sufficient evidence to prefer ATM-AVI over meropenem in MBL positive infections [53].

## 6. Cefepime–Taniborbactam

Cefepime–taniborbactam combines the fourth-generation cephalosporin cefepime with taniborbactam, a bicyclic boronate β-lactamase inhibitor [54]. Unlike other available inhibitors, taniborbactam demonstrates potent inhibitory activity against Ambler class B metallo-β-lactamases (MBLs), as well as class A, C, and D serine β-lactamases (including KPC, OXA-48, ESBLs, and AmpC). In vitro studies have confirmed its robust activity against carbapenem-resistant *Enterobacterales* (CRE) and carbapenem-resistant *Pseudomonas aeruginosa*, including strains co-producing NDM, VIM, KPC, and other carbapenemases [55].

### Clinical Evidence

The Phase 3 CERTAIN-1 trial, a randomized, double-blind, multicenter study, evaluated cefepime–taniborbactam versus meropenem for the treatment of complicated urinary tract infections (cUTIs), including acute pyelonephritis. Among 661 randomized patients, 436 were included in the microbiological intention-to-treat (microITT) population. The primary endpoint—composite success (microbiological eradication plus clinical resolution) at the test-of-cure visit—was achieved in 70.6% (207/293) of patients receiving cefepime–taniborbactam compared to 58.0% (83/143) with meropenem, demonstrating superiority (treatment difference: 12.6%; 95% CI: 3.1–22.2; *p* = 0.009). This benefit was sustained through late follow-up (63.8% vs. 51.7%). Furthermore, in the extended microITT population, composite success was achieved in 8 of 10 patients (80%) with meropenem-resistant pathogens, including 6 of 7 (85.7%) with *K. pneumoniae* [56]. Safety profiles were comparable, with adverse events occurring in 35.5% of the cefepime–taniborbactam group and 29.0% of the meropenem group. Most events were mild to moderate in severity; serious adverse events were reported in 2.0% and 1.8% of patients, respectively.

While recently published intrapulmonary pharmacokinetic data provide a strong rationale for cefepime–taniborbactam, their direct clinical applicability to severe infections caused by MBL-producing *Enterobacterales* remains limited. Phase 1 data show that a 4-h infusion achieves epithelial lining fluid (ELF) penetration ratios of 0.153–0.253 for taniborbactam, supporting further investigation in pneumonia due to susceptible MDR Gram-negative pathogens [57]. However, the pivotal CERTAIN-1 trial, which demonstrated clinical efficacy, included only patients with complicated urinary tract infections and excluded those with bloodstream infections (BSIs) or pneumonia [56]. Moreover, the subgroup with confirmed MBL-producing pathogens was not characterized, and the number of meropenem-resistant cases was insufficient to draw MBL-specific conclusions. Therefore, despite encouraging pharmacokinetic findings, randomized clinical data supporting the efficacy of cefepime–taniborbactam in BSI or pneumonia are currently lacking.

## 7. Fosfomycin

Fosfomycin is a bactericidal phosphonic acid derivative that inhibits UDP-NN-acetylglucosamine enolpyruvyl transferase (MurA), the enzyme catalyzing the first committed step of peptidoglycan biosynthesis [58].

Clinical evidence supporting intravenous fosfomycin for carbapenem-resistant Gram-negative infections is derived primarily from observational studies. In a prospective multinational cohort of 716 patients treated across five European countries, Gram-negative pathogens were frequently identified, including *Klebsiella* spp. (17.2%) and *E. coli* (14.2%). Carbapenem resistance was present in 48.0% of *Klebsiella* spp. isolates. Fosfomycin was administered predominantly as combination therapy (90.2%), with a median daily dose of 15 g. Among 77 patients with carbapenem-resistant infections—mainly due to *Klebsiella* spp.—clinical success was achieved in 81.8% and microbiological cure in 83.1%. In the subgroup with bacteraemia (*n* = 22), both outcomes were observed in 86.4% of patients. Overall in-hospital mortality was 10.6%, with no deaths attributed to fosfomycin. However, the non-comparative design limits interpretation, as most patients received concomitant active agents, most commonly ceftazidime–avibactam, precluding attribution of outcomes to fosfomycin [59].

Additional data from a prospective observational study by Falcone et al. provide insight into MBL-producing *Enterobacterales*. Among 343 patients, fosfomycin susceptibility was 67.1%, and 22 received fosfomycin-containing regimens. Thirty-day mortality in this subgroup was 18.2%. However, these patients had substantially lower baseline severity than those treated with ceftazidime–avibactam plus aztreonam, including lower SOFA scores, fewer bloodstream infections (13.6% vs. 64.7%), minimal ICU admission, and no septic shock. Most infections were urinary tract in origin, and all cases of secondary bacteraemia had adequate source control. After adjustment, no significant difference in 30-day mortality was observed between treatment groups (18.2% vs. 22.3%, *p* = 0.791), indicating that crude outcome differences likely reflect baseline characteristics rather than treatment effect [60].

Overall, available data suggest that intravenous fosfomycin, primarily used in combination regimens, may be associated with favorable outcomes in selected patients with carbapenem-resistant Gram-negative infections, particularly of urinary origin. However, in the absence of randomized controlled trials in MBL-producing *Enterobacterales*, its role remains uncertain. Given the high risk of confounding by indication and preferential use in less severe, non-bacteraemic infections, fosfomycin should currently be considered an adjunctive rather than a primary therapeutic option [61].

## 8. Colistin (Polymyxin E) and Polymyxin B

Colistin (polymyxin E) is a cationic polypeptide antibiotic that exerts bactericidal activity against *Enterobacterales* by binding to lipid A in the lipopolysaccharide (LPS) of the outer membrane. By displacing divalent cations, colistin destabilizes the LPS layer and disrupts membrane integrity. Polymyxin B shares the same mechanism of action but differs pharmacokinetically, as it is administered as the active compound rather than as the prodrug colistimethate sodium, resulting in more predictable plasma exposure. However, comparative clinical data between colistin and polymyxin B in *Enterobacterales* infections remain limited [62,63].

Clinical evidence supporting colistin-based therapy in MBL-producing *Enterobacterales* is derived mainly from small observational studies. In a study of 31 patients with carbapenem-resistant Gram-negative infections treated with ceftazidime–avibactam plus colistin, carbapenem-resistant *K. pneumoniae* was the predominant pathogen (54.8%, 17/31). MBL production was identified in 7 patients (22.6%; IMP-type, 4; NDM-type, 3). Within this subgroup, 30-day mortality was 28.6% (2/7), with both deaths occurring in patients with NDM-producing *K. pneumoniae* and baseline septic shock. Microbiological clearance within 7 days was achieved in 71.4% (5/7) [64].

Comparative data between ceftazidime–avibactam-based and polymyxin-based regimens in carbapenem-resistant *K. pneumoniae* infections have also emerged. In a propensity score-matched multicenter study of 276 patients with CR-KP infections, ceftazidime–avibactam-based therapy was associated with higher clinical efficacy (71.3% vs. 56.1%; *p* = 0.011), improved microbiological clearance (74.7% vs. 41.4%; *p* < 0.001), and a lower incidence of acute kidney injury (13.5% vs. 33.7%; *p* < 0.001) than polymyxin B-based regimens. These findings support the current treatment paradigm in which polymyxins, including colistin and polymyxin B, are reserved for situations in which newer β-lactam/β-lactamase inhibitor combinations are unavailable, inactive, or contraindicated [65].

A multicenter propensity score-matched study by Zhuang et al. demonstrated that polymyxin B-based treatment achieved a clinical success rate of approximately 69% in severe carbapenem-resistant Gram-negative infections, although nephrotoxicity remained common, occurring in approximately one-third of treated patients. These findings further support the role of polymyxins primarily as salvage or alternative agents when preferred β-lactam-based options are unavailable [66].

Overall, colistin and polymyxin B retain in vitro activity against many carbapenem-resistant *Enterobacterales* and may be used in combination regimens [67]. However, their clinical utility is limited by dose-dependent nephrotoxicity, inferior outcomes compared with newer agents in comparative studies, and the absence of randomized trials demonstrating non-inferiority. Current IDSA and ESCMID guidance positions polymyxins as salvage agents for MBL-producing *Enterobacterales* infections when first-line options, including ceftazidime–avibactam plus aztreonam where available, cannot be used [3,4]. Clinicians should also be cautious when interpreting isolated reports of emerging resistance, as robust population-level surveillance is needed to define clinically meaningful trends in polymyxin susceptibility among *Enterobacterales*.

## 9. Antibiotic Adjuvants: Emerging Strategies and Future Directions

Antibiotic adjuvants are small molecules with limited intrinsic antibacterial activity that enhance the efficacy of existing antibiotics by targeting bacterial resistance mechanisms. Although most research has focused on β-lactamase inhibition, other adjuvant strategies are attracting increasing interest, including agents that interfere with efflux pumps, biofilm formation, and essential bacterial pathways [68]. Among these, inhibition of bacterial cystathionine γ-lyase (bCSE), an enzyme implicated in bacterial survival and virulence, represents a particularly novel approach [69]. Collectively, these developments highlight the expanding therapeutic scope of antibiotic adjuvants and their potential to preserve the utility of existing antimicrobials. Within this landscape, however, metallo-β-lactamase inhibitors (MBLIs) remain one of the most urgent unmet needs in antimicrobial drug development [70].

The global spread of MBL-producing pathogens, particularly those carrying NDM, VIM, and IMP enzymes, underscores the need for inhibitors capable of restoring the activity of existing β-lactams. Unlike serine-β-lactamase inhibitors, which have achieved clinical success, no MBLI has yet been approved. This gap reflects the structural diversity of MBL active sites, the complexity of zinc-dependent catalysis, and the challenge of developing compounds with broad-spectrum activity and favorable pharmacokinetic properties [71].

Several structurally distinct candidates have shown encouraging preclinical activity. ANT2681, a sulfonamide-based inhibitor, preferentially inhibits NDM-1 and has restored meropenem activity in murine infection models caused by NDM-1-producing *K. pneumoniae* [72,73]. Xeruborbactam is a boron-based inhibitor with activity against both serine and metallo-β-lactamases and is currently in clinical development [74]. KSP-1007, a bicyclic boronate inhibitor, has completed Phase I evaluation in combination with meropenem and has demonstrated broad-spectrum in vitro and in vivo activity against carbapenem-resistant Gram-negative pathogens. Together, these agents support the feasibility of pharmacological MBL inhibition and provide a foundation for further optimization [75]. Synthetic antibiotic adjuvants are widely integrated into clinical practice and have demonstrated proven efficacy [76]. The development of MBL inhibitors (MBLIs) constitutes a critical therapeutic strategy to overcome bacterial resistance mechanisms and preserve the effectiveness of existing antibiotics, marking a significant advancement in addressing the AMR crisis.

## 10. Discussion

The management of infections caused by MBL-producing *Enterobacterales* remains one of the most difficult problems in contemporary clinical microbiology and infectious diseases. These pathogens are associated with substantial morbidity and mortality, and therapeutic decisions are complicated by limited susceptibility to most conventional antimicrobial classes. In this setting, the most clinically relevant options are currently cefiderocol and ceftazidime–avibactam plus aztreonam, with polymyxin-based regimens and tigecycline largely relegated to salvage or resource-limited settings [53].

The combination of ceftazidime–avibactam plus aztreonam (CAZ-AVI + ATM) exploits complementary vulnerabilities: avibactam inhibits co-produced serine β-lactamases (e.g., ESBLs, KPC, AmpC) that would otherwise hydrolyze both ceftazidime and aztreonam, while aztreonam itself is inherently stable against MBLs and provides the bactericidal activity against MBL-producing *Enterobacterales*. Practical therapeutic decision rule: where aztreonam–avibactam (ATM–AVI) is available and susceptibility is confirmed, it should be considered the preferred targeted treatment for infections caused by MBL-producing *Enterobacterales*. Where ATM–AVI is unavailable, ceftazidime–avibactam plus aztreonam (CAZ–AVI + ATM) remains the preferred alternative and is supported by current IDSA and ESCMID recommendations [3,4].

The evidence base for CAZ-AVI + ATM is stronger than for many older regimens but remains largely observational. Available studies consistently suggest favorable outcomes, particularly in bloodstream infection and severe disease caused by MBL-producing organisms, yet the absence of randomized controlled trials limits the certainty of inference [77].

Cefiderocol also remains an important treatment option, but its role is nuanced. Although it retains activity against many carbapenem-resistant Gram-negative pathogens and has demonstrated clinical utility in selected resistant infections, susceptibility is less reliable in MBL-producing strains, especially those harboring NDM enzymes. This variability means cefiderocol should ideally be guided by susceptibility testing and local epidemiology rather than used as a universal solution for all MBL producers. Its place in therapy is therefore complementary rather than definitive, particularly in centers with access to more targeted β-lactam/β-lactamase inhibitor combinations [3].

By contrast, tigecycline and fosfomycin have a more limited role. Tigecycline may retain utility in selected infections, especially when used in combination therapy, but safety concerns, low serum concentrations, and inconsistent outcome data limit its appeal for severe bloodstream infections. Fosfomycin is promising as an adjunctive agent, especially in urinary or less severe infections, but the current clinical evidence remains too heterogeneous and confounded to support it as a primary therapy for MBL-producing *Enterobacterales*. These agents are therefore best viewed as alternatives when preferred options are unavailable or unsuitable [78,79].

Polymyxins, including colistin and polymyxin B, have historically served as salvage agents, but their role is increasingly diminished. Although they retain in vitro activity against some carbapenem-resistant isolates, nephrotoxicity, inferior comparative outcomes, and the absence of high-quality randomized evidence argue against routine use when newer agents are accessible. Their remaining value lies mainly in settings where preferred β-lactam-based therapies are unavailable, inactive, or contraindicated [63].

The future therapeutic landscape may broaden further with the development of cefepime–taniborbactam and other MBL-directed inhibitors. Cefepime–taniborbactam is notable because it is one of the few investigational agents with activity against MBLs as well as serine β-lactamases, and early clinical data are encouraging for complicated urinary tract infection [80,81]. However, its current evidence base is not yet sufficient to establish a clear role in bloodstream infection or pneumonia caused by MBL-producing organisms. More broadly, the development of MBL inhibitors remains a critical unmet need, because the approved β-lactamase inhibitor class still does not reliably address class B enzymes [82].

Early identification of patients at risk of clinical deterioration remains essential. In this context, the LIP score proposed by Liu et al. may assist in the early recognition of severe CRE infections and support timely escalation of appropriate antimicrobial therapy [83].

Treatment selection should not rely solely on the resistance mechanism. Clinical factors, particularly the site of infection, remain critical because bloodstream infections, hospital-acquired or ventilator-associated pneumonia, complicated urinary tract infections, and intra-abdominal infections may require different pharmacokinetic and pharmacodynamic considerations.

From a stewardship perspective, the main clinical message is that treatment should be individualized according to pathogen identity, resistance mechanism, infection site, severity, and local drug availability. In all cases, susceptibility testing, molecular characterization, and access to expert infectious diseases input remain essential for optimizing outcomes [48,84].

## 11. Conclusions

Infections caused by MBL-producing *Enterobacterales* continue to present a major therapeutic challenge. Among currently available options, ceftazidime–avibactam plus aztreonam is emerging as the most targeted and clinically attractive treatment where accessible. Cefiderocol retains an important role but should be used selectively, given variable activity against MBL producers, especially NDM-harboring isolates [3,47].

Older agents such as tigecycline, fosfomycin, and polymyxins remain useful only in selected circumstances and should generally be reserved for salvage or combination strategies when preferred agents cannot be used [85]. The pipeline of MBL inhibitors, including cefepime–taniborbactam and other investigational compounds, offers promise for expanding future therapeutic choices, but randomized clinical data are still needed [86]. Overall, the current evidence supports a mechanism-based, stewardship-informed approach in which ceftazidime–avibactam plus aztreonam occupies a central role in the treatment of confirmed MBL-producing *Enterobacterales* infections [86].

## Data Availability

No new data were created or analyzed in this study.

## Abbreviations

The following abbreviations are used in this manuscript:

BAT	best available therapy
cUTI	complicated urinary tract infection
CRP	C-reactive protein
ESBL	extended-spectrum β-lactamase
HAP	hospital-acquired pneumonia
HSCT	hematopoietic stem cell transplantation
IAI	intra-abdominal infection
ITT	intent-to-treat
LPS	lipopolysaccharide
MBL	metallo-β-lactamase
MDR	multidrug-resistant
MIC	minimum inhibitory concentration
NDM	New Delhi metallo-β-lactamase
SOFA	sequential organ failure assessment
UTI	urinary tract infection
VAP	ventilator-associated pneumonia
